# Discoid Lupus Erythematosus-Associated Cutaneous Squamous Cell Carcinoma in Systemic Lupus Erythematosus

**DOI:** 10.5152/eurasianjmed.2022.21062

**Published:** 2022-06-01

**Authors:** Meltem Alkan Melikoglu, Mehmet Melikoglu, Elif Demirci, Ensar Zafer Barin

**Affiliations:** 1 Ataturk University School of Medicine, Physical Medicine and Rehabilitation, Rheumatology Department, Erzurum, Turkey; 2 Ataturk University School of Medicine, Dermatology, Erzurum, Turkey; 3 Ataturk University School of Medicine, Pathology, Erzurum, Turkey; 4 Ataturk University School of Medicine, Aesthetic, Plastic and Reconstructive Surgery, Erzurum, Turkey

Dear Editor,

Systemic lupus erythematosus (SLE) is a multisystem disease with a large range of manifestations.^1^ Several cutaneous manifestations can be seen in the disease course. Discoid lupus erythematosus (DLE) is the most common type of cutaneous lupus erythematosus. It may be associated with an increased risk of cutaneous squamous cell carcinoma (cSCC) that may have a poorer prognosis in cases with particular risk factors. The aim of this manuscript was to share our patient with SLE presenting cSCC as a complication of DLE and to draw attention to these risk factors in such cases.

A 50-year-old woman with a history of SLE and DLE for 8 years has presented a non-healing cutaneous ulcerated lesion over the discoid area on her nose (Figure 1). Due to the persistent ulcers for 6 months in the DLE area, a skin biopsy was performed. As dysplastic changes and atypical cells were seen in the histopathological evaluation of skin biopsy, surgical excision was performed. The histopathological evaluation of the excision materal was consistent with the cSCC diagnosis (Figure 2). Her previous treatment regimen for SLE (hydroxychloroquine and azathioprine) has remained and no re-occurrence in cSCC has been noted during 12 month-follow-up of the patient. Written informed consent for the images was obtained from the patient.

Discoid lupus erythematosus is characterized by scarring lesions with pigmentary changes mostly on sun-exposed skin areas.^2^ High-risk skin cancers are a rare, but severe, complication of DLE. Cutaneous squamous cell carcinoma has been reported with an incidence up to 3% of patients with DLE.^2^ Cutaneous squamous cell carcinoma may present different clinical behaviors ranging from relatively non-aggressive forms to aggressive ones with significant metastatic potential, but scars and immunosuppression have been reported as predisposing factors associated with high-risk subtypes.^3^ Previous studies have reported poorer data in cSCC associated with DLE with higher rates of recurrence and metastasis than sporadic cSCC cases.^4^ Also a 4-fold higher risk of cSCC development has been reported in patients with DLE which is associated with a 20-fold increase in mortality compared to patients with sporadic cSCC (2). So the clinical course of cSCC as a complication of DLE may be aggressive, with early metastases and risk of mortality.^5,6^

Although the pathophysiology underlying the development of DLE-associated cSCC has not been defined, several factors may cause predisposition to skin cancer in DLE. Cutaneous malignancies have been significantly associated with autoimmune connective tissue diseases (ACTDs) including SLE, systemic sclerosis, dermatomyositis, and Sjögren syndrome due to several potential pathogenetic mechanisms such as chronic scar, ultraviolet (UV) radiation, inflammation, immunosuppressive treatments, viral infections, and smoking.^7^ Long-standing discoid lesions seem to be one of the major factors in cSCC arising in DLE since previously described cases generally presented long-term DLE history as in our case.^6^ Also UV radiation is a well-known risk factor for cSCC and DLE lesions located preferentially on sun-exposed body areas.^2^ Disease-related immune system impairment and cutaneous inflammation are also suggested as possible pathogenetic mechanisms.^7^ Immunosuppressives have been considered as factors promoting cSCC development; however, similar cSCC prevalence was reported between DLE patients with or without immunosuppressive drug history (2). Although the underlying pathophysiology has not been clarified yet, DLE-associated cSCC seems to be reasonable in patients with ACTDs because of several cumulative risk factors in this disease spectrum. Also, it has been stated that the clinical presentation may be challenging and the cSCC risk airing in DLE can be minimized by early anti-inﬂammatory treatment.^8,9^

In conclusion, cSCC, one of the most common form of non-melanotic skin cancer, may develop as a complication of DLE, especially in patients who have additional risk factors due to the nature of the co-incident diseases such as ACTDs. The poorer prognosis of cSCC in DLE patients highlights the need for better screening strategies for early detection and prompt therapeutic interventions.

## Figures and Tables

**Figure 1.**
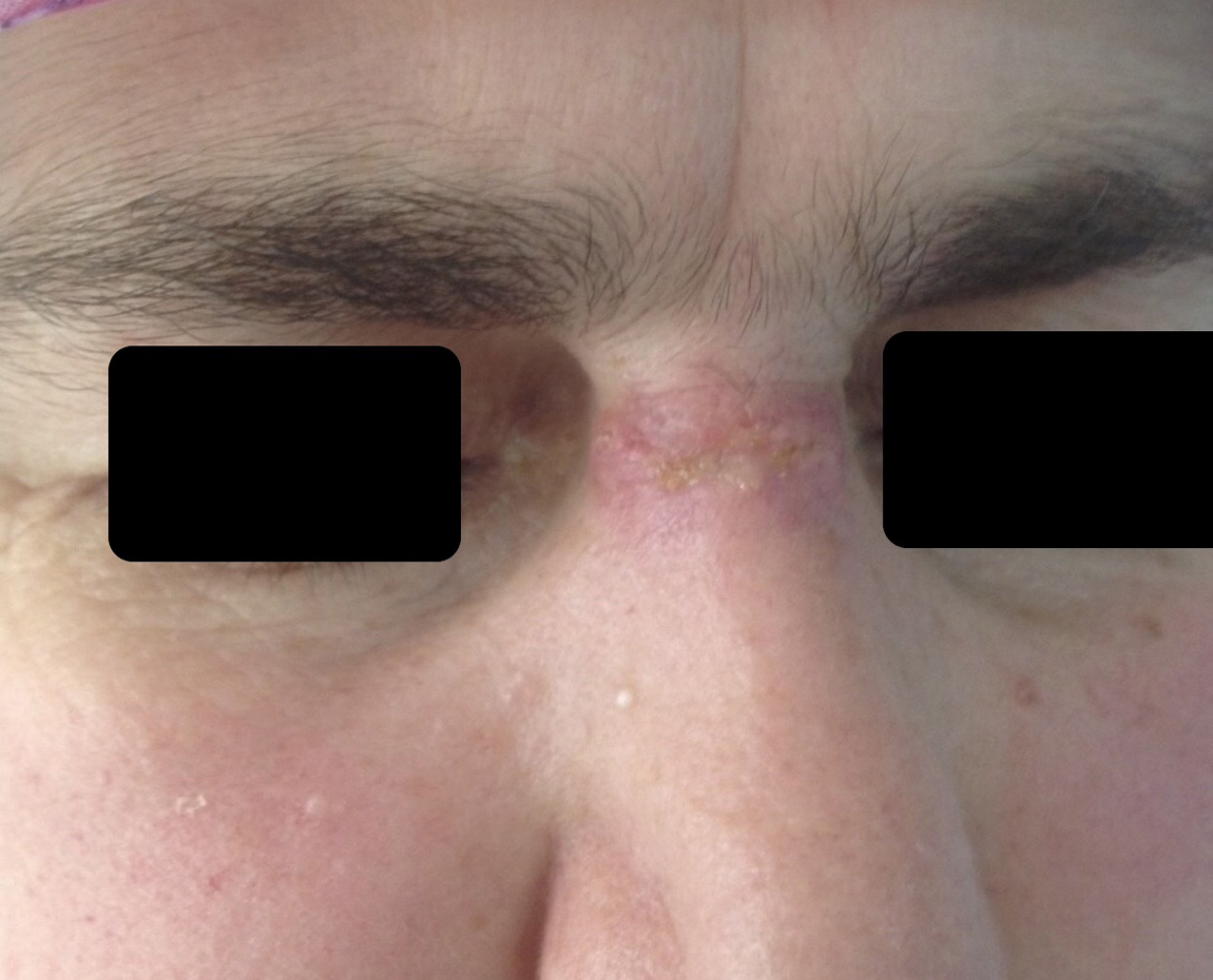
Cutaneous ulcerated lesion over the discoid area on the nose.

**Figure 2.**
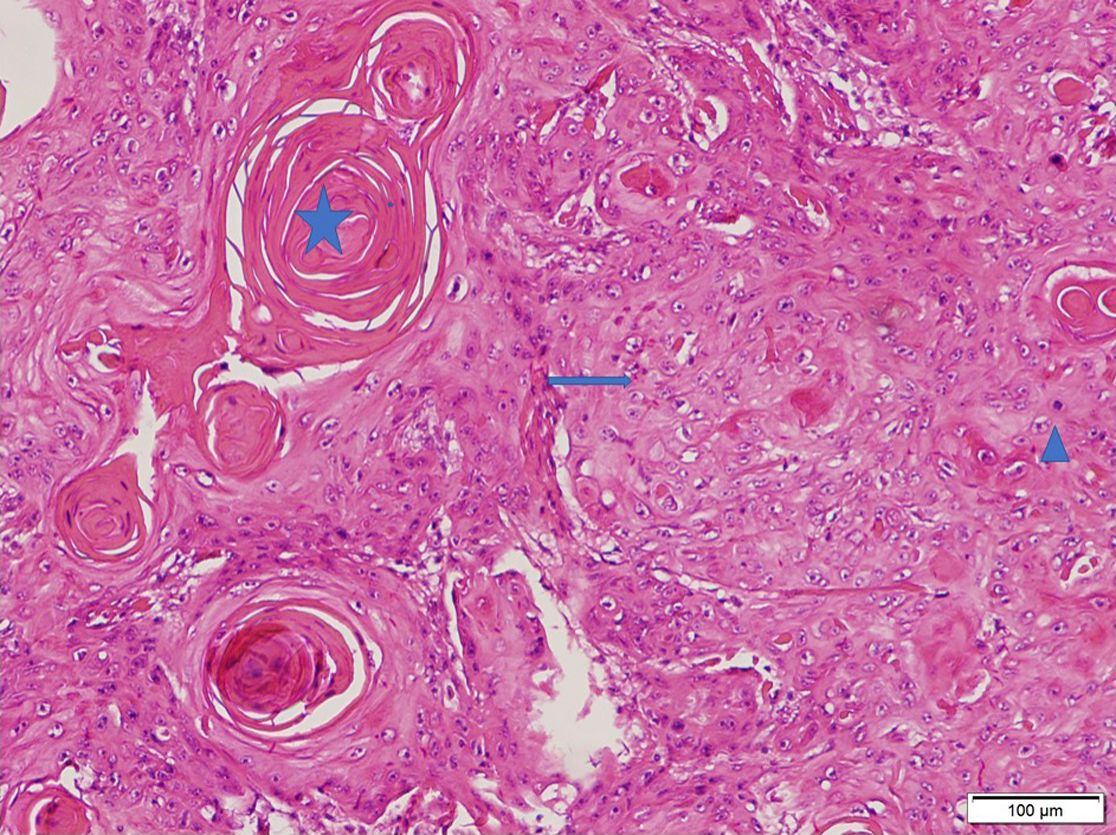
The histopathological evaluation of the excision materal was consistent with cSCC diagnosis; HE X100 star: Easily recognizable squamous epithelium, abundant keratinization arrow: minimal pleomorphism arrow head: mitotic figures. cSCC, cutaneous squamous cell carcinoma.
